# Investigação da Relação entre Índice de Triglicerídeos-Glicose e Fluxo Lento Coronariano: Um Estudo Retrospectivo de Caso-Controle

**DOI:** 10.36660/abc.20220679

**Published:** 2023-05-16

**Authors:** Mustafa Kaplangoray, Kenan Toprak, Fuat Başanalan, Ali Palice, Cihan Aydın, Aykut Demirkıran, Yusuf Cekici

**Affiliations:** 1 Departamento de Cardiologia University of Health Sciences Mehmet Akif İnan Research and Training Hospital Sanlıurfa Turquia Departamento de Cardiologia – University of Health Sciences Mehmet Akif İnan Research and Training Hospital , Sanlıurfa – Turquia; 2 Republic of Turkey Ministry Health Siverek State Hospital Cardiology Department Sanlıurfa Turquia Republic of Turkey Ministry of Health Siverek State Hospital Cardiology Department , Sanlıurfa – Turquia; 3 Departamento de Cardiologia Tekirdag Namık Kemal University Tekirdağ Turquia Departamento de Cardiologia , Tekirdag Namık Kemal University , Tekirdağ – Turquia; 4 Departamento de Cardiologia University of Health Sciences Adana Health Practice Research Center Adana Turquia Departamento de Cardiologia , University of Health Sciences Adana Health Practice and Research Center , Adana – Turquia

**Keywords:** Doença Arterial Coronária, Velocidade do Fluxo Sanguíneo, Triglicerides-Glicose, Lipoproteínas, LDL, Isquemia Miocárdica

## Abstract

**Fundamento:**

O fluxo lento coronariano (FLC) refere-se à opacificação retardada dos vasos distais na ausência de estenose da artéria coronária epicárdica. O mecanismo etiopatogênico do FLC ainda não está claro.

**Objetivos:**

Este estudo investiga a relação entre o FLC e o índice de triglicerídeos-glicose (TyG).

**Métodos:**

A amostra do estudo consistiu de 118 pacientes com FLC e 105 pacientes com fluxo coronariano normal (FCN). A taxa de fluxo coronariano foi medida por medio do método de contagem de quadros (TFC) Thrombolysis in Myocardial Infarction (TIMI) em todos os pacientes. O índice TyG foi calculado como o logaritmo do valor [triglicerídeos em jejum (mg/dL)×glicose em jejum (mg/dL)]/2. Adotou-se como estatisticamente significativo o nível de significância < 0,05.

**Resultados:**

O índice TyG, lipoproteína de baixa densidade (LDL), índice de massa corporal (IMC), relação neutrófilo-linfócito (RNL) e valores de TFC, proporção masculina e proporção de fumantes foram maiores, enquanto os níveis de lipoproteína de alta densidade (HDL) foram significativamente menores no grupo FLC em comparação com o grupo FNC (p<0,05). A análise de correlação revelou que o FLC estava significativamente correlacionado com os valores do índice TyG, IMC, RNL e HDL. A mais forte dessas correlações foi entre o FLC e o índice TyG (r= 0,57, p<0,001). Além disso, a análise multivariada revelou que o índice TyG, IMC, razão RNL e sexo masculino foram preditores independentes para FLC (p<0,05). A análise da curva ROC (Receiver Operating Characteristic) indicou que um valor de corte ≥ 9,28 para o índice TyG previu FLC com sensibilidade de 78% e especificidade de 78,1% [Área sob a curva (AUC): 0,868 e 95% intervalo de confiança (IC): 0,823-0,914].

**Conclusão:**

Os achados deste estudo revelaram uma relação muito forte entre o FLC e o índice TyG.

**Figura Central:**
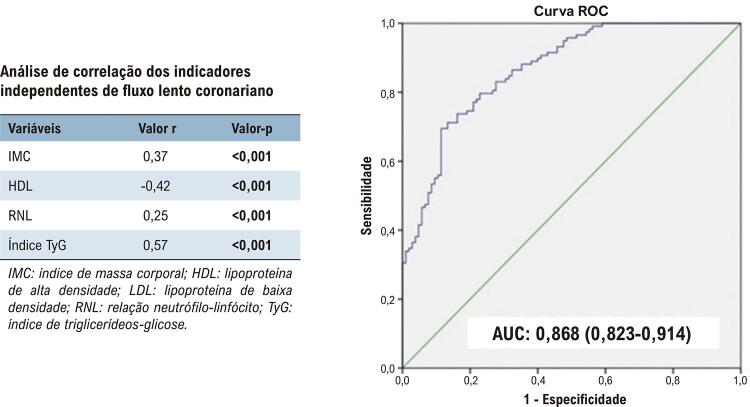
: Investigação da Relação entre Índice de Triglicerídeos-Glicose e Fluxo Lento Coronariano: Um Estudo Retrospectivo de Caso-Controle

## Introdução

O fenômeno de fluxo lento coronariano (FLC) refere-se à opacificação tardia das artérias coronárias epicárdicas normais. A incidência de FLC em pacientes submetidos à angiografia coronária (CAG) para queixas de angina é relatada entre 1-3%. ^1 , 2^ Embora estudos em larga escala tenham sido conduzidos para descobrir sua etiologia e mecanismo subjacente, a etiologia e a patogênese do FLC ainda não estão claras. Entre os fatores mais frequentemente responsabilizados pela patogênese do FLC estão disfunção endotelial, anormalidades microvasculares, aterosclerose oculta e inflamação. ^3 , 4^ A relação entre FLC e índice de massa corporal (IMC), níveis de glicose, dislipidemia e síndrome metabólica (SM) foi demonstrada em um estudo de larga escala conduzido por Yılmaz et al., ^5^ Os achados relevantes de muitos estudos clínicos e experimentais sugerem que a disfunção endotelial desempenha um papel importante na patogênese do FLC. ^6^

A relação entre resistência à insulina e doença arterial coronariana (DAC) está bem estabelecida. No entanto, essa relação não foi claramente demonstrada no contexto da PSC. Os achados relevantes relatados pelos estudos disponíveis na literatura são contraditórios. ^7 - 9^ Em um desses estudos, Metwally et al. ^7^ demonstraram a relação entre resistência à insulina e FLC em indivíduos com intolerância à glicose. O índice triglicerídeo-glicose (TyG) é um parâmetro facilmente calculado e barato que reflete a resistência à insulina. No entanto, uma revisão completa da literatura não revelou nenhum estudo que abordasse a relação entre o FLCP e o índice TyG. Portanto, este estudo foi desenhado para investigar a relação entre o FLC e o índice TyG.

## Métodos

O presente estudo foi um estudo observacional, caso-controle e comparativo. Aproximadamente 3.000 participantes submetidos à angiografia coronária diagnóstica eletiva em nossa instituição foram escaneados para identificar pacientes com FLC aparente. A indicação de CAG foi determinada com base na angina pectoris típica e suas queixas características ou na positividade dos testes de estresse não invasivos realizados para investigação de isquemia miocárdica. Os critérios de exclusão do estudo foram: ter evoluído com FLC secundário a angioplastia coronária percutânea realizada após infarto do miocárdio ou cirurgia de revascularização miocárdica; ter uma doença cardíaca valvar orgânica significativa, insuficiência cardíaca congestiva, doença cardíaca congênita, fibrilação atrial, hipo/hipertireoidismo ou qualquer distúrbio do tecido conjuntivo; possuir alguma doença hematológica;)ou insuficiência hepática [aspartato transaminase (AST) ou alanina transaminase (ALT) valores 3 vezes superiores aos valores normais]; ter uma infecção ativa; e/ou faz uso de drogas do grupo das estatinas e/ou anticoagulantes. Os prontuários médicos do paciente foram digitalizados retrospectivamente em relação aos critérios de exclusão do estudo. Ao final, 118 pacientes com FLC e 105 pacientes com fluxo coronariano normal (FCN) foram incluídos na amostra do estudo. Dados demográficos referentes à idade, sexo e fatores de risco para DAC (hipertensão, diabetes, dislipidemia, história familiar e tabagismo) foram obtidos do banco de dados do hospital. Além disso, a bioquímica de rotina, os resultados do hemograma e os parâmetros de glicemia e colesterol em jejum foram determinados a partir dos resultados de sangue obtidos dos registros dos pacientes antes do CAG. O índice TyG foi calculado como o logaritmo do valor [triglicerídeos em jejum (mg/dL)×glicose em jejum (mg/dL)]/2. A relação neutrófilo-linfócito (RNL) foi calculada dividindo-se a contagem absoluta de neutrófilos medida com as amostras de sangue coletadas na admissão pela contagem absoluta de linfócitos.

A fração de ejeção do ventrículo esquerdo (FEVE) foi obtida a partir de registros ecocardiográficos antes da CAG. Os pacientes que receberam tratamento anti-hipertensivo foram considerados hipertensos, e o diagnóstico de hiperlipidemia foi definido de acordo com os critérios diagnósticos das diretrizes da European Society of Cardiology. ^10^ Os diabéticos foram determinados por pacientes que já haviam sido diagnosticados com diabetes e faziam uso de medicamentos antidiabéticos e outros pacientes que não conheciam seu estado de diabetes, mas tinham glicose alta no sangue de acordo com os critérios da American Diabetes Association. ^11^ O estudo foi conduzido de acordo com os princípios da Declaração de Helsinque, e o protocolo do estudo foi aprovado pelo Comitê de Ética da Faculdade de Medicina da Harran University (Número: HRÜ/22.16.11).

### Cateterismo cardíaco

O procedimento CAG foi realizado por via femoral ou radial pela técnica de Judkins. Artéria coronária normal foi definida como a ausência de irregularidade do lúmen em qualquer artéria coronária com base na avaliação visual. As contagens de quadros coronarianos dos pacientes foram calculadas de acordo com o cálculo de contagem de quadros (TFC) Thrombolysis in Myocardial Infarction (TIMI), conforme descrito por Poyraz et al., ^8^ o cálculo do TFC foi feito a partir da angulação caudal oblíqua anterior direita ou projeção da angulação craniana oblíqua anterior esquerda para as artérias descendente anterior esquerda (DAE) e circunflexa (Cx), e da projeção oblíqua anterior esquerda para a artéria coronária direita (CD). O primeiro quadro em que o meio de contraste cobriu todo o lúmen da artéria coronária proximal, tocou ambas as bordas e se moveu para baixo na artéria foi aceito como o primeiro quadro. Em contraste, o quadro em que o material opaco atingiu a região do bigode para DAE, a bifurcação distal com a maior distância total do ramo marginal do obtuso marginal para Cx e o primeiro ramo lateral emergindo da artéria póstero-lateral para CD foram aceitos como último quadro. O valor de TFC de cada artéria foi calculado subtraindo o número do primeiro quadro do último. Considerando o tempo decorrido para a opacificação devido ao comprimento da DAE, o TFC corrigido foi calculado dividindo-se o valor do TFC calculado para DAE por 1,7. Conforme relatado anteriormente na literatura, os valores corrigidos do limite de TFC foram aceitos como 36,2 ± 2,6 quadros para DAE, 22,2 ± 4,1 quadros para a artéria Cx e 20,4 ± 3,0 quadros para a artéria CD ^8^ . Pacientes com valores de TFC maiores que 2 desvios padrão dos valores limiares especificados em qualquer uma das três artérias foram diagnosticados com FLC.

### Análise estatística

As análises estatísticas foram realizadas por meio do pacote de software SPSS 22.0 (Statistical Package for Social Sciences for Windows, versão 22.0, IBM Corp, Armonk, NY, EUA, 2013). Os dados com distribuição normal foram expressos como média ± desvio padrão (DP), e as variáveis categóricas foram expressas como frequências absolutas e relativas. O teste de Kolmogorov-Smirnov foi utilizado para determinar as características normais das variáveis contínuas. O teste t de amostras independentes foi usado para comparar as variáveis contínuas determinadas para obedecer à distribuição normal. O teste qui-quadrado de Pearson ou teste exato de Fisher foi usado para comparar as variáveis categóricas. A análise da curva ROC (Receiver Operating Characteristic) foi realizada para determinar os valores de corte do índice TyG que podem ser usados na previsão de FLCP.

Testes de análise de regressão logística univariada e multivariada foram realizados para identificar os preditores independentes de FLCP. Valores de probabilidade (p) < 0,05 indicaram significância estatística. O coeficiente de correlação de Pearson foi utilizado para a análise de correlação entre os indicadores independentes do FLCP.

## Resultados

As características demográficas, comorbidades, resultados de exames laboratoriais e uso de medicamentos de ambos os grupos estão resumidos na Tabela 1 . Assim, a média de idade dos pacientes incluídos no estudo foi de 51,7±9,4 anos, e 57,4% dos pacientes incluídos no estudo eram homens. Não houve diferença significativa entre os grupos de pacientes e controle em relação às comorbidades, a saber, hiperlipidemia, DM e HT. Por outro lado, o valor médio do IMC e a proporção de fumantes foram maiores no grupo de pacientes do que no grupo controle. Os níveis médios de triglicerídeos, LDL, RNL e glicose foram significativamente maiores no grupo de pacientes do que no grupo de controle.

**Tabela 1:** – Características demográficas e clínicas da população estudada

	Grupo FNC (n=105)	Grupo CSF (n=118)	Total (n=223)	Valor-p
**Demografia**				
Idade (ano)	51,0±9,7	52,2±9,2	51,7±9,4	0,343
Masc., (%)	37 (35,2%)	91 (77,1%)	128 (57,4%)	**<0,001**
**Comorbidades**				
IMC (kg/m ^2^ )	25,2±2,9	27,6±3,1	26,5±3,2	**<0,001**
Hipertensão n (%)	34 (32,4%)	45 (38,1%)	79 (35,4%)	0,370
Hiperlipidemia n (%)	36 (34,3%)	56 (52,5%)	92 (41,3%)	0,056
D.Mellitus n (%)	25 (23,8%)	37 (31,4%)	62 (23,3%)	0,209
Uso de nicotina n (%)	33 (31,4%)	74 (62,7%)	107 (48%)	**<0,001**
**Medições quantitativas**				
PAS (mmHg)	125,8±15,4	129±17,4	127,5±16,5	0,130
PAD (mmHg)	75,4±10,8	77,5±12,1	76,5±11,5	0,270
Glicose (mg/dl)	111,2±29	162,9±18,8	138,6±82,9	**<0,001**
T. Colesterol (mg/dl)	216±33,4	222±35,6	219,66±34,7	0,138
Triglicerídeos (mg/dl)	167,3±42,9	222,9±60,9	196,78±59,9	**<0,001**
HDL (mg/dl)	45,7±11,6	37,4±6,1	41,2±9,9	**<0,001**
LDL (mg/dl)	133,7±29,6	142,2±22,9	138,2±26,6	**0,018**
Leucócitos (×103/µL)	8,3±1,8	9,4±0,8	8,9±0,7	0,216
Neutrófilo (×103/µL)	4,4±1,2	5,4±2,1	4,9±1,8	**<0,001**
Linfócito (×103/µL)	2,4±0,8	2,3±0,8	2,3±0,8	0,438
RNL	2,2±1,2	2,6±1,2	2,4±1,2	**0,010**
Monócito (×103/µL)	0,7±0,8	1,2±0,7	0,9±0,4	0,403
Plaquetas (×103/µL)	274,3±86,3	244,5±65,1	258,5±77,1	0,08
Hemoglobina (g/dl)	13,6±1,9	14,5±1,6	14,1±1,8	0,07
Creatinina (mg/dl)	1,0±0,9	0,9±0,1	0,9±0,7	0,278
BUN (mg/dl)	30,7±10,3	30±9,9	29,3±10,1	0,588
TyG	9,1±0,4	9,7±0,5	9,4±0,5	**<0,001**
FE, (%)	61,5±2,9	60,3±3,9	60,8±5,2	0,580
**Uso de medicamentos**				
Aspirina n(%)	22 (21,2%)	43 (36,4%)	65 (29,3%)	**0,012**
B-Bloqueador n (%)	15(14,4%)	29 (24,6%)	44 (19,8%)	0,058
CCB n (%)	15(14,3%)	17(14,4%)	32 (14,3%)	0,979
IECA/BRA n (%)	28 (26,9%)	36 (30,5%)	64 (28,8%)	0,556
**Dados angiográficos**				
DAE FC	19,6±5,8	40,3±10,0	30,7±13,5	**0,001**
LCX FC	17,1±4,6	28,0±6,9	22,8±8,7	**0,001**
CD FC	18,7±6,8	32,6±9,4	26,0±10,9	**0,001**

*IMC: índice de massa corporal; PAS: pressão arterial sistólica; PAD: pressão arterial diastólica; HDL: lipoproteína de alta densidade; LDL: lipoproteína de baixa densidade; RNL: relação neutrófilo-linfócito; TyG: índice de triglicerídeos-glicose; CCB: canal de cálcio bloqueador; IECA: inibidor da enzima conversora de angiotensina; BRA: bloqueador do receptor de angiotensina; DAE: artéria descendente anterior esquerda; LCX: artéria circunflexa; CD: artéria coronária direita; FC: Contagem de quadros TIMI corrigida; GB: contagem de glóbulos brancos.*

Por outro lado, o nível médio de HDL foi significativamente maior no grupo controle do que no grupo de pacientes. Não houve diferença significativa entre os grupos quanto aos medicamentos utilizados, exceto a aspirina, que foi significativamente mais utilizada no grupo de pacientes do que no grupo de controle. As contagens de quadros TIMI foram maiores no grupo de pacientes do que no grupo de controle para todas as três artérias. Os valores médios do índice TyG do grupo de pacientes foram significativamente maiores do que os do grupo controle. Os resultados da análise de correlação estão resumidos na Tabela 2 . Consequentemente, o FLC foi positivamente correlacionado com os valores do índice de IMC, RNL e TyG e negativamente correlacionado com os valores de HDL. Além disso, houve uma correlação positiva moderada significativa entre os valores de TyG e FLC.

**Tabela 2:** – Análise de correlação dos indicadores independentes de fluxo lento coronariano

Variáveis	Valor r	Valor-p
IMC	0,37	**<0,001**
HDL	-0,42	**<0,001**
RNL	0,25	**<0,001**
Índice TyG	0,57	**<0,001**

*IMC: índice de massa corporal; HDL: lipoproteína de alta densidade; LDL: lipoproteína de baixa densidade; RNL: relação neutrófilo-linfócito; TyG: índice de triglicerídeos-glicose.*

A análise de regressão logística univariada revelou uma relação significativa entre FLC e IMC, HDL, triglicerídeos, glicose, RNL, valores do índice TyG, proporção masculina, e a proporção de fumantes. Uma análise mais aprofundada dessas variáveis usando a análise de correlação multivariada revelou que os valores de índice de IMC, RNL, TyG e proporção masculina foram preditores independentes de FLC ( Tabela 3 ).

**Tabela 3:** – Análises de regressão logística univariada e multivariada dos indicadores independentes de fluxo coronariano lento

	Análise univariada		Análise multivariada	
Variáveis	OU (IC 95%)	Valor de p	OU (IC 95%)	Valor de p
IMC	0,767 (0,694-0,848)	<0,001	1.309 (1.145-1.496)	**<0,001**
HDL	1.131 (1.082-1.182)	<0,001	0,954 (0,904-1,007)	0,086
Triglicerídeo	1.030 (1.020-1.040)	<0,001	-	-
Glicose	1.020 (1.012-1.029)	<0,001	-	-
Gênero	6.19 (3.443-11.143)	<0,001	3.497 (1.501-8.147)	**0,004**
Fumo	3.669 (2.105-6.632)	<0,001	0,454 (0,201-1,024)	0,057
RNL	0,734 (0,575-0,938)	0,013	1.407 (1.032-1.919)	0,031
TyG	31.489 (2.966-33.429)	<0,001	27.649 (8.044-95.035)	**<0,001**

*IC: intervalo de confiança; IMC: índice de massa corporal; HDL: lipoproteína de alta densidade; LDL: lipoproteína de baixa densidade; RNL: relação neutrófilo-linfócito; TyG: índice de triglicerídeos-glicose.*

**Figura 1:**
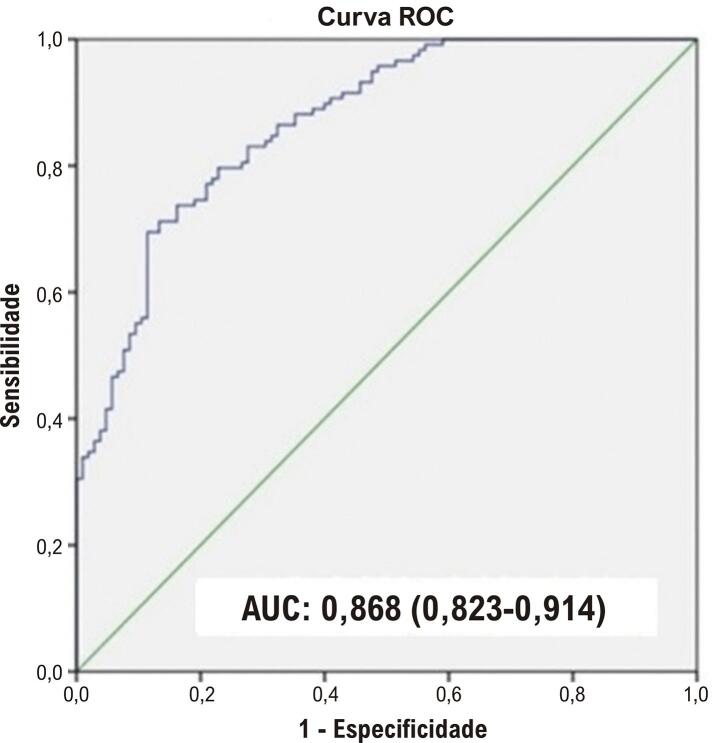
– Análise ROC dos níveis do índice TyG para prever a circulação de fluxo lento coronariano. AUC: Área sob a curva; ROC: Característica operacional do receptor; TyG: índice de triglicérides-glicose.

A análise da curva ROC indicou que um valor de corte ≥ 9,28 para o índice TyG previu FLC com sensibilidade de 78% e especificidade de 78,1% [Área sob a curva (AUC): 0,868 e IC de 95%: 0,823-0,914] ( Figura 1 ).

## Discussão

O FLC é um importante achado angiográfico observado em pacientes com síndrome coronariana aguda, principalmente se apresentarem queixa de angina instável. O FLCP deve ser considerado uma entidade clínica distinta, dados seus mecanismos clínicos e patogênicos específicos e critérios diagnósticos estabelecidos. Estudos anteriores mostraram que a doença de pequenos vasos sanguíneos, disfunção endotelial, aterosclerose subclínica e inflamação são os fatores implicados na patogênese do FLC. ^2^ O endotélio desempenha um papel fundamental no desenvolvimento da ativação plaquetária, adesão de leucócitos, proliferação de células vasculares e processo de aterosclerose, particularmente no equilíbrio do tônus vascular. Pacientes com FLC têm redução da dilatação mediada por fluxo (DMF) dependente do endotélio na artéria braquial. Este achado suporta a hipótese de que a disfunção endotelial desempenha um papel na etiologia do FLC. ^3^ Além disso, acredita-se sem dúvida que a disfunção endotelial e a resistência à insulina desempenham um papel fundamental em todas as fases do desenvolvimento da aterosclerose. ^12 , 13^ Em um estudo recente, Metwally et al., ^7^ demonstraram que a resistência à insulina é um fator independente para o FLC em pacientes com intolerância à glicose. A Avaliação do Modelo Homeostático para Resistência à Insulina (HOMA-IR) e o clamp hiperinsulinêmico-euglicêmico são frequentemente usados para medir a resistência à insulina. ^14^ No entanto, a medição da resistência à insulina com base no nível de insulina não é um método frequentemente utilizado na prática clínica diária, uma vez que é caro e de difícil acesso na maioria dos laboratórios. Por outro lado, foi demonstrado em vários estudos que o índice TyG pode ser usado no lugar da resistência à insulina como uma alternativa barata, prática e reprodutível. ^15^ Além disso, vários estudos mostraram que o índice TyG fornece resultados confiáveis na determinação da resistência à insulina por meio de HOMA IR e avaliação do teste clamp hiperinsulinêmico-euglicêmico. ^14^

No entanto, uma revisão completa da literatura não revelou nenhum estudo que abordasse a relação entre o FLC e o índice TyG. Nesse contexto, este estudo foi desenhado para investigar a relação entre o FLC e o índice TyG. Consequentemente, os achados deste estudo indicaram uma forte relação entre o FLC e o índice TyG. A relação entre o FLC e os níveis de glicose e hiperlipidemia foi demonstrada em estudos anteriores. No entanto, os resultados desses estudos são contraditórios. ^16^ Em comparação, a relação entre o FLC e o índice TyG foi estatisticamente mais significativa do que a relação entre o FLC e os dois parâmetros mencionados, a saber, níveis de glicose e hiperlipidemia, neste estudo. A análise de correlação também indicou que a relação entre o FLC e o índice TyG foi significativamente mais forte do que a relação entre o FLC e os valores de IMC, HDL e RNL ( Tabela 2 ).

Além disso, a análise de regressão multivariada revelou que os valores de IMC, HDL e RNL, proporção de homens e proporção de fumantes são importantes preditores independentes de FLC, além do índice TyG. Este achado é consistente com o achado relatado no estudo de Zhao et al., ^6^ que os valores de IMC e proporção masculina são preditores independentes de FLC. Da mesma forma, Arbel et al., ^17^ relataram que a proporção de homens e a proporção de fumantes são fortes preditores de FLC. ^17^ Em contraste, Sanghvi et al., ^16^ não encontraram uma relação significativa entre a proporção masculina e o FLC. ^16^

Os achados deste estudo indicaram uma forte relação entre FLC e RNL. Foi demonstrado em muitos estudos que o FLC pode ser uma manifestação de doença aterosclerótica difusa. ^18 , 19^ Além disso, foi estabelecido recentemente que a doença aterosclerótica é um processo inflamatório crônico. ^20 - 23^ Portanto, a inflamação é uma característica importante e manifestação clínica de aterosclerose. O papel da inflamação na aterosclerose foi demonstrado em numerosos estudos. ^24 - 26^ A contagem de glóbulos brancos (GB) e RNL, um derivado de GB, são marcadores de inflamação sistêmica. De fato, os achados deste estudo revelaram que a RNL é um fator forte e independente para o FLC.

### Limitações do estudo

Houve algumas limitações para este estudo devido ao seu desenho. Primeiro, a população do estudo era relativamente pequena. Em segundo lugar, alguns achados laboratoriais e demográficos dos pacientes não puderam ser alcançados devido à natureza retrospectiva do estudo e, portanto, a relação entre FLC e índice TyG no contexto dos referidos achados laboratoriais não pôde ser investigada. Em terceiro lugar, a correlação entre o índice TyG considerado um marcador de resistência à insulina e o nível de insulina não pôde ser investigado, uma vez que os níveis de insulina dos pacientes não puderam ser acessados.

## Conclusão

Embora muitos fatores desempenhem um papel na etiopatogenia do FLC, a disfunção endotelial está entre os mais importantes. A resistência à insulina desempenha um papel importante na disfunção endotelial. Classicamente, o método “padrão-ouro” para avaliar a sensibilidade à insulina é o teste clamp hiperinsulinêmico-euglicêmico. No entanto, esse teste é caro e demorado, portanto seu uso prático é limitado. Recentemente, o índice TyG tem sido usado frequentemente como um indicador de resistência à insulina. Este estudo revelou uma relação muito forte entre o FLC e o índice TyG. No entanto, mais estudos são necessários para apoiar o uso do índice TyG como um preditor independente para FLC.
