# Supplementary material for: Investigação da Relação entre Índice de Triglicerídeos-Glicose e Fluxo Lento Coronariano: Um Estudo Retrospectivo de Caso-Controle
Source: Arq Bras Cardiol. 2023 May 16;120(6):e20220679. [Article in Portuguese] doi: 10.36660/abc.20220679 (PMC10389274; doi:10.36660/abc.20220679)
Supplement: Translation

# Translation: Investigation of the Relationship Between Triglycerides-Glucose İndex and Coronary Slow Flow: A Retrospective Case-Control Study

**Authors:** Mustafa Kaplangoray, Kenan Toprak, Fuat Başanalan, Ali Palice, Cihan Aydın, Aykut Demirkıran, Yusuf Cekici

**Affiliations:** 1 Turkey Departamento de Cardiologia – University of Health Sciences Mehmet Akif İnan Research and Training Hospital, Sanlıurfa – Turkey; 2 Turkey Republic of Turkey Ministry of Health Siverek State Hospital Cardiology Department, Sanlıurfa – Turkey; 3 Turkey Departamento de Cardiologia, Tekirdag Namık Kemal University, Tekirdağ – Turkey; 4 Turkey Departamento de Cardiologia, University of Health Sciences Adana Health Practice and Research Center, Adana – Turkey

**Keywords:** Coronary Artery Disease, Blood Flow Velocity, Triglicerideos-Glucose, Lipoproteins, LDL, Myocardial Ischemic

## Abstract

**Background:**

Coronary slow flow (CSF) refers to delayed distal vessel opacification in the absence of epicardial coronary artery stenosis. The etiopathogenic mechanism of CSF is still unclear.

**Objectives:**

This study investigates the relationship between CSF and the triglyceride-glucose (TyG) index.

**Methods:**

The study sample consisted of 118 CSF patients and 105 patients with normal coronary flow (NCF). The coronary flow rate was measured via the Thrombolysis in Myocardial Infarction (TIMI) frame count (TFC) method in all patients. The TyG index was calculated as the logarithm of the [fasting triglyceride (mg/dL)×fasting glucose (mg/dL)]/2 value. A significance level of < 0.05 was adopted as statistically significant.

**Results:**

The TyG index, low-density lipoprotein (LDL), body mass index (BMI), neutrophil-to-lymphocyte ratio (NLR) and TFC values, male ratio, and the ratio of smokers were higher, whereas high-density lipoprotein (HDL) levels were significantly lower in the CSF group compared to the NCF group (p<0,05). The correlation analysis revealed that CSF was significantly correlated with TyG index, BMI, NLR, and HDL values. The strongest of these correlations was between CSF and TyG index (r= 0.57, p<0.001). Additionally, the multivariate analysis revealed that TyG index, BMI, NLR ratio, and male gender were independent predictors for CSF (p<0.05). Receiver operating characteristic (ROC) curve analysis indicated that a cut-off value of ≥ 9.28 for the TyG index predicted CSF with a sensitivity of 78% and a specificity of 78.1% [Area under the curve (AUC): 0.868 and 95% Confidence Interval (CI): 0.823-0.914].

**Conclusion:**

The findings of this study revealed a very strong relationship between CSF and TyG index.

## Introduction

Coronary slow flow phenomenon (CSF) refers to delayed opacification of normal epicardial coronary arteries. The incidence of CSF in patients who underwent coronary angiography (CAG) for anginal complaints is reportedly between 1-3%.^1 , 2^Although large-scale studies have been conducted to uncover its etiology and underlying mechanism, the etiology and pathogenesis of CSF are still unclear. Among the most frequently blamed factors for the pathogenesis of CSF are endothelial dysfunction, microvascular abnormalities, occult atherosclerosis, and inflammation.^3 , 4^The relationship between CSF and body mass index (BMI), glucose levels, dyslipidemia, and metabolic syndrome (MS) has been demonstrated in a large-scale study conducted by Yılmaz et al.^5^The relevant findings of many clinical and experimental studies suggest that endothelial dysfunction plays an important role in the pathogenesis of CSF.^6^

The relationship between insulin resistance and coronary artery disease (CAD) is well established. Nevertheless, this relationship has not been clearly demonstrated in the context of CSF. The relevant findings reported by the studies available in the literature are contradictory.^7 - 9^In one of these studies, Yaser et al.^7^demonstrated the relationship between insulin resistance and CSF in individuals with impaired glucose tolerance. The triglyceride-glucose (TyG) index is an easily calculated and inexpensive parameter that reflects insulin resistance. However, a thorough literature review did not reveal any study that addressed the relationship between CSFP and TyG index. Therefore, this study has been designed to investigate the relationship between the CSF and the TyG index.

**Central Illustration:** : Investigation of the Relationship Between Triglycerides-Glucose İndex and Coronary Slow Flow: A Retrospective Case-Control Study

## Methods

The present study was an observational, case–control, and comparative study. Approximately 3000 participants who underwent elective diagnostic coronary angiography at our institution were scanned to identify patients with apparent CSF. Indication for CAG was determined based on typical angina pectoris and its characteristic complaints or the positive results of the noninvasive stress tests performed to investigate myocardial ischemia. The study exclusion criteria were as follows: having developed with CSF secondary to percutaneous coronary angioplasty performed after myocardial infarction or coronary bypass surgery; having a significant organic valvular heart disease, congestive heart failure, congenital heart disease, atrial fibrillation, hypo/hyperthyroidism or any connective tissue disorder; having any hematological disease; having any autoimmune and neoplastic disease, chronic renal (eGFR less than 60 mL/min/1.73 m2 or dialysis) or hepatic insufficiency [aspartate transaminase (AST) or alanine transaminase (ALT) values 3 times higher than normal values]; having an active infection; and/or have been using statin group drugs and/or anticoagulants. The patient’s medical files were retrospectively scanned against the study exclusion criteria. In the end, 118 CSF patients and 105 patients with normal coronary flow (NCF) were included in the study sample. Demographic data regarding age, gender, and risk factors for CAD (hypertension, diabetes, dyslipidemia, family history, and cigarette smoking) were obtained from the hospital database. In addition, routine biochemistry, hemogram results, and fasting blood glucose and cholesterol parameters were determined from the blood results obtained from the patient records before CAG. The TyG index was calculated as the logarithm of the [fasting triglyceride (mg/dL)×fasting glucose (mg/dL)]/2 value. The neutrophil-to-lymphocyte ratio (NLR) was calculated by dividing the absolute neutrophil count measured using the blood samples collected at admission by the absolute lymphocyte count.

Left ventricular ejection fraction (LVEF) was obtained from echocardiographic recordings before CAG. Patients receiving antihypertensive treatment were considered hypertensive patients, and the diagnosis of hyperlipidemia was defined according to the diagnostic criteria of the European Society of Cardiology guidelines.^10^Diabetics were determined by patients who had already been diagnosed with diabetes and taking antidiabetic medications and other patients who did not know their diabetes status but had high blood glucose according to the American Diabetes Association’s criteria.^11^The study was conducted following the principles of the Helsinki Declaration, and the study protocol was approved by the Harran University Faculty of Medicine Ethics Committee (Number: HRÜ/22.16.11).

### Cardiac catheterization

The CAG procedure was performed via the femoral or radial route using the Judkins technique. Normal coronary artery was defined as the absence of lumen irregularity in any coronary artery based on the visual evaluation. Patients’ coronary frame counts were calculated according to the Thrombolysis in Myocardial Infarction (TIMI) frame count (TFC) calculation, as described by Poyraz et al.^8^TFC calculation was made from right anterior oblique caudal angulation or left anterior oblique cranial angulation projection for left anterior descending (LAD) and circumflex (Cx) arteries, and from the left anterior oblique projection for the right coronary artery (RCA). The first frame in which the contrast medium covered the entire proximal coronary artery lumen, touched both borders and moved down the artery was accepted as the first frame. In contrast, the frame in which the opaque material reached the mustache region for LAD, the distal bifurcation with the longest total distance of the marginal branch of the marginal obtuse for Cx, and the first lateral branch emerging from the posterolateral artery for RCA were accepted as the last frame. Each artery’s TFC value was calculated by subtracting the first frame number from the last. Considering the time elapsed for opacification due to LAD length, corrected TFC was calculated by dividing the TFC value calculated for LAD by 1.7. As previously reported in the literature, the corrected TFC threshold values were accepted as 36.2 ± 2.6 frames for the LAD, 22.2 ± 4.1 frames for the Cx artery and 20.4 ± 3.0 frames for the RCA artery^8^. Patients with TFC values greater than 2 standard deviations from the threshold values specified in any of the three arteries were diagnosed with CSF.

### Statistical analysis

The statistical analyses were performed using the software package SPSS 22.0 (Statistical Package for Social Sciences for Windows, version 22.0, IBM Corp, Armonk, NY, U.S., 2013). Data with normal distribution were expressed as mean ± standard deviation (SD), and categorical variables were expressed as absolute and relative frequencies. The Kolmogorov-Smirnov test was used to determine the normal characteristics of the continuous variables. The independent-sample t-test was used to compare the continuous variables determined to conform to the normal distribution. Pearson’s chi-squared or Fisher’s exact test was used to compare the categorical variables. Receiver operating characteristic (ROC) curve analysis was performed to determine the cut-off values of the TyG index that can be used in the prediction of CSFP.

Univariate and multivariate logistic regression analysis tests were performed to identify the independent predictors of CSFP. Probability (p) values of < 0.05 indicated statistical significance. Pearson’s correlation coefficient was used for the correlation analysis between the independent indicators of CSFP.

## Results

Both groups’ demographic characteristics, comorbidities, laboratory test results, and medication use are summarized in Table 1 . Accordingly, the mean age of the patients included in the study was 51.7±9.4 years, and 57.4% of the patients included in the study were male. There was no significant difference between the patient and control groups regarding comorbidities, namely hyperlipidemia, DM, and HT. On the other hand, the mean BMI value and ratio of smokers were higher in the patient group than in the control group. The mean triglyceride, LDL, NLR, and glucose levels were significantly higher in the patient group than in the control group.

**Table 1:** – Demographic and clinical characteristics of the study population

	NCF group (n=105)	CSF group (n=118)	Total (n=223)	p-value
**Demographics**				
Age (year)	51.0±9.7	52.2±9.2	51.7±9.4	0.343
Male, (%)	37 (35.2%)	91 (77.1%)	128 (57.4%)	**<0.001**
**Comorbidities**				
BMI (kg/m^2^)	25.2±2.9	27.6±3.1	26.5±3.2	**<0.001**
Hypertension n (%)	34 (32.4%)	45 (38.1%)	79 (35.4%)	0.370
Hyperlipidemia n (%)	36 (34.3%)	56 (52.5%)	92 (41.3%)	0.056
D.Mellitus n (%)	25 (23.8%)	37 (31.4%)	62 (23.3%)	0.209
Nikotine use n (%)	33 (31.4%)	74 (62.7%)	107 (48%)	**<0.001**
**Quantitative measurements**				
SBP (mmHg)	125.8±15.4	129±17.4	127.5±16.5	0.130
DBP (mmHg)	75.4±10.8	77.5±12.1	76.5±11.5	0.270
Glucose (mg/dl)	111.2±29	162.9±18.8	138.6±82.9	**<0.001**
T. Cholesterol (mg/dl)	216±33.4	222±35.6	219.66±34.7	0.138
Triglyceride (mg/dl)	167.3±42.9	222.9±60.9	196.78±59.9	**<0.001**
HDL (mg/dl)	45.7±11.6	37.4±6.1	41.2±9.9	**<0.001**
LDL (mg/dl)	133.7±29.6	142.2±22.9	138.2±26.6	**0.018**
WBC (×103/µL)	8.3±1.8	9.4±0.8	8.9±0.7	0.216
Neutrophil (×103/µL)	4.4±1.2	5.4±2.1	4.9±1.8	**<0.001**
Lymphocyte (×103/µL)	2.4±0.8	2.3±0.8	2.3±0.8	0.438
NLR	2.2±1.2	2.6±1.2	2.4±1.2	**0.010**
Monocyte (×103/µL)	0.7±0.8	1.2±0.7	0.9±0.4	0.403
Platelet (×103/µL)	274.3±86.3	244.5±65.1	258.5±77.1	0.08
Hemoglobin (g/dl)	13.6±1.9	14.5±1.6	14.1±1.8	0.07
Creatinin (mg/dl)	1.0±0.9	0.9±0.1	0.9±0.7	0.278
BUN (mg/dl)	30.7±10.3	30±9.9	29.3±10.1	0.588
TyG	9.1±0.4	9.7±0.5	9.4±0.5	**<0.001**
EF, (%)	61.5±2.9	60.3±3.9	60.8±5.2	0.580
**Medication usage**				
Aspirin n(%)	22 (21.2%)	43 (36.4%)	65 (29.3%)	**0.012**
B-Blocker n (%)	15(14.4%)	29 (24.6%)	44 (19.8%)	0.058
CCB n (%)	15(14.3%)	17(14.4%)	32 (14.3%)	0.979
ACEİ/ARB n (%)	28 (26.9%)	36 (30.5%)	64 (28.8%)	0.556
**Angiographic data**				
LAD FC	19.6±5.8	40.3±10.0	30.7±13.5	**0.001**
LCX FC	17.1±4.6	28.0±6.9	22.8±8.7	**0.001**
RCA FC	18.7±6.8	32.6±9.4	26.0±10.9	**0.001**

*BMI: body mass index; SBP: systolic blood pressure; DBP: diastolic blood pressure; HDL: high-density lipoprotein; LDL: low-density lipoprotein; NLR: neutrophil-lymphocyte ratio; TyG: triglyceride-glucose index; CCB: calcium channel blocker; ACEI: angiotensin converting enzyme inhibitor; ARB: angiotensin receptor blocker; LAD: left anterior descending artery; LCX: circumflex artery; RCA: right coronary artery; FC: corrected TIMI frame count; WBC: white blood cell count.*

On the other hand, the mean HDL level was significantly higher in the control group than in the patient group. There was no significant difference between the groups in medications used, except for aspirin, which was found to be used significantly more in the patient group than in the control group. TIMI frame counts were higher in the patient group than in the control group for all three arteries. The mean TyG index values of the patient group were significantly higher than that of the control group. The results of the correlation analysis are summarized in Table 2 . Accordingly, CSF was positively correlated with BMI, NLR, and TyG index values and negatively correlated with HDL values. In addition, there was a significant moderate positive correlation between TyG values and CSF.

**Table 2:** – Correlation analysis of the independent indicators of coronary slow flow

Variables	r value	p-value
BMI	0.37	**<0.001**
HDL	-0.42	**<0.001**
NLR	0.25	**<0.001**
TyG index	0.57	**<0.001**

*BMI: body mass index; HDL: high-density lipoprotein; NLR: neutrophil-lymphocyte ratio; TyG: triglyceride-glucose index.*

The univariate logistic regression analysis revealed a significant relationship between CSF and BMI, HDL, triglyceride, glucose, NLR, TyG index values, male ratio, and the ratio of smokers. Further analysis of these variables using the multivariate correlation analysis revealed that BMI, NLR, TyG index values, and male ratio were independent predictors of CSF ( Table 3 ).

**Table 3:** – Univariate and multivariate logistic regression analyses of the independent indicators of slow coronary flow

	Univariate analysis		Multivariate analysis	
Variables	OR (95% CI)	p- value	OR (95% CI)	p- value
BMI	0.767 (0.694-0.848)	<0.001	1.309 (1.145-1.496)	**<0.001**
HDL	1.131 (1.082-1.182)	<0.001	0.954 (0.904-1.007)	0.086
Triglyceride	1.030 (1.020-1.040)	<0.001	-	-
Glucose	1.020 (1.012-1.029)	<0.001	-	-
Gender	6.19 (3.443-11.143)	<0.001	3.497 (1.501-8.147)	**0.004**
Smoking	3.669 (2.105-6.632)	<0.001	0.454 (0.201-1.024)	0.057
NLR	0.734 (0.575-0.938)	0.013	1.407 (1.032-1.919)	0.031
TyG	31.489 (2.966-33.429)	<0.001	27.649 (8.044-95.035)	**<0.001**

*CI: confidence interval; BMI: body mass index; HDL: high-density lipoprotein; NLR: neutrophil-lymphocyte ratio; TyG: triglyceride-glucose index.*

ROC curve analysis indicated that a cut-off value of ≥ 9.28 for the TyG index predicted CSF with a sensitivity of 78% and a specificity of 78.1% [Area under the curve (AUC): 0.868 and 95% CI: 0.823-0.914] ( Figure 1 ).

**Figure 1:** – ROC analysis of the TyG index levels for predicting slow flow coronary circulation. AUC: Area under the curve; ROC: Receiver operating characteristic; TyG: triglyceride-glucose index.

## Discussion

CSF is an important angiographic finding observed in acute coronary syndrome patients, particularly if presenting with the complaint of unstable angina. CSFP should be considered a distinct clinical entity given its specific clinical and pathogenic mechanisms and established diagnostic criteria. Previous studies have shown that small blood vessel disease, endothelial dysfunction, subclinical atherosclerosis, and inflammation are the factors implicated in the pathogenesis of CSF.^2^Endothelium plays a key role in developing platelet activation, leukocyte adhesion, vascular cell proliferation, and atherosclerosis process, particularly in vascular tone balance. Patients with CSF reportedly have reduced endothelium-dependent flow-mediated dilation (FMD) in the brachial artery. This finding supports the hypothesis that endothelial dysfunction plays a role in the etiology of CSF.^3^In addition, it is believed without a doubt that endothelial dysfunction and insulin resistance play a key role at every stage of the development of atherosclerosis.^12 , 13^In a recent study, Metwally et al.^7^demonstrated that insulin resistance is an independent factor for CSF in patients with impaired glucose tolerance. Homeostatic Model Assessment for Insulin Resistance (HOMA-IR) and hyperinsulinemic-euglycemic clamp test are frequently used to measure insulin resistance.^14^However, measurement of insulin resistance based on insulin level is not a frequently resorted method in daily clinical practice since it is expensive and difficult to access in most laboratories. On the other hand, it has been shown in numerous studies that the TyG index can be used in place of insulin resistance as an inexpensive, practical, and reproducible alternative.^15^In addition, numerous studies have shown that the TyG index provides reliable results in determining insulin resistance through HOMA IR and hyperinsulinemic-euglycemic clamp test assessment.^14^

However, a thorough literature review did not reveal any study that addressed the relationship between CSF and TyG index. In this context, this study has been designed to investigate the relationship between CSF and TyG index. Consequentially, the findings of this study indicated a strong relationship between CSF and TyG index. The relationship between CSF and glucose levels and hyperlipidemia has been demonstrated in previous studies. Nevertheless, the results of these studies are contradictory.^16^In comparison, the relationship between CSF and TyG index was found to be statistically more significant than the relationship between CSF, and the said two parameters, namely glucose levels and hyperlipidemia, in this study. The correlation analysis also indicated that the relationship between CSF and TyG index was significantly stronger than the relationship between CSF and BMI, HDL, and NLR values ( Table 2 ).

Furthermore, the multivariate regression analysis revealed that BMI, HDL, and NLR values, male ratio, and the ratio of smokers are important independent predictors of CSF, in addition to the TyG index. This finding is consistent with the finding reported in Zhao et al.’s study^6^that BMI values and male ratio are independent predictors of CSF. Similarly, Arbel et al.^17^reported that the male ratio and the ratio of smokers are strong predictors of CSF.^17^In contrast, Sanghvi et al.^16^did not find a significant relationship between the male ratio and CSF.^16^

The findings of this study indicated a strong relationship between CSF and NLR. It has been demonstrated in many studies that CSF can be a manifestation of diffuse atherosclerotic disease.^18 , 19^Additionally, it has been established recently that atherosclerotic disease is a chronic inflammatory process.^20 - 23^Hence, inflammation is an important feature and clinical manifestation of atherosclerosis. The role of inflammation in atherosclerosis has been demonstrated in numerous studies.^24 - 26^White blood cell (WBC) count and NLR, a derivative of WBC, are markers of systemic inflammation. As a matter of fact, the findings of this study revealed that NLR is a strong and independent factor for CSF.

### Study limitations

There were some limitations to this study due to its design. First, the study population was relatively small. Secondly, some laboratory and demographic findings of the patients could not be reached due to the study’s retrospective nature, and hence the relationship between CFS and TyG index in the context of the said laboratory findings could not be investigated. Thirdly, the correlation between the TyG index considered a marker of insulin resistance, and the insulin level could not be investigated since the patients’ insulin levels could not be accessed.

## Conclusion

Although many factors play a role in the etiopathogenesis of CSF, endothelial dysfunction is among the most important. Insulin resistance plays an important role in endothelial dysfunction. Classically, the “gold standard” method for evaluating insulin sensitivity is the hyperinsulinemic-euglycemic clamp test. However, this test is expensive and time-consuming, so its practical use is limited. Recently, the TyG index has been used frequently as an indicator of insulin resistance. This study revealed a very strong relationship between CSF and TyG index. However, further studies are needed to support using the TyG index as an independent predictor for CSF.
